# Novel Insights into Addiction Management: A Meta-Analysis on Intervention for Relapse Prevention

**DOI:** 10.3390/medicina61040619

**Published:** 2025-03-28

**Authors:** Dana Cătălina Tabugan, Ana Cristina Bredicean, Teodora Anghel, Raluca Dumache, Camelia Muresan, Leonardo Corsaro, Lavinia Hogea

**Affiliations:** 1 Neuroscience Department, “Victor Babes” University of Medicine and Pharmacy, 300041 Timisoara, Romania; dana.tabugan@umft.ro (D.C.T.); anghel.teodora@umft.ro (T.A.); raluca.dumache@umft.ro (R.D.); amelia.muresan@umft.ro (C.M.); hogea.lavinia@umft.ro (L.H.); 2 Neuropsychology and Behavioral Medicine Center, “Victor Babes” University of Medicine and Pharmacy, 300041 Timisoara, Romania; 3 Ethics in Human Genetic Identification Center, “Victor Babes” University of Medicine and Pharmacy, 300041 Timisoara, Romania; 4 Campus Bio Medico, 00128 Roma, Italy; leo_corsaro@hotmail.it

**Keywords:** addiction, relapse prevention, pharmacological intervention, non-pharmacological intervention, detoxification

## Abstract

*Background and Objectives*: Addiction and relapse prevention of alcohol and drug users is a real problem globally. Studies report different pharmacological and non-pharmacological methods in preventing relapse with varying ranges of results across the time of relapse. The study aims to identify novel insights into relapse prevention for high-risk alcohol and drug addiction across diverse global populations, ages, and intervention types during detoxification. *Materials and Methods*: This meta-analysis followed PRISMA guidelines, synthesizing 12 eligible studies published between 2013 and 2023, totaling 2162 participants. Data extraction and statistical analysis were conducted using Python-based libraries. Regression models were applied to examine the influence of age, gender, and intervention type on the mean relapse period. *Results*: 12 studies with 2162 patients were identified. These studies examined substances, interventions, and demographics, highlighting male predominance in addictive behaviors. OSL regression assessed factors influencing mean relapse periods, finding that age explained 44.2% of the variability (*p* = 0.0131). The male percentage explained 17.1%, but the significance was inconclusive, as was the female gender’s negligible impact (14.7% variability). Intervention types significantly influenced relapse periods, supported by a large F-statistic. Linear regression showed no consistent trend in relapse periods, with declining research post-2018. Forest plots indicated disparities in relapse periods due to treatment or methodology. Most participants were high-risk drug users, though alcohol use was also represented. A declining trend in publication rates after 2018 was observed. *Conclusions*: Age and intervention type were identified as key factors influencing relapse duration, while gender and substance-specific effects require further study. The findings underscore the need for more targeted, gender-sensitive, and context-aware treatment strategies.

## 1. Introduction

Addiction is defined as a chronic, relapsing brain disorder. Substance misuse is a significant global issue, particularly in developed countries. The most commonly abused substances are alcohol and illicit drugs [1]. In 2020, an estimated 284 million people (5.6%) aged 15–67 had used a drug in the last 12 months [2,3]. This fact represents a 26% increase compared to 2010 [4]. Global estimates of drug users include 209 million for cannabis, 61 million for opioids, 34 million for amphetamines, and 20 million for cocaine and ecstasy [4]. The World Health Organization (WHO) estimated that 283 million people had alcohol use disorders worldwide in 2016 [1]. The most dangerous substance is opioids, which are the leading cause of drug overdose deaths, as tolerance decreases after a period of abstinence during the relapse phase [5,6,7]. Relapse rates for substance use, ranging from 40% to 93% within the first six months after treatment, highlight the need for relapse-sensitive care and additional treatment methods [1].

Relapse in substance use is a concept applied across all disciplines in health and behavioral science, particularly in the field of addiction. It refers to a return to substance use after an individual has previously managed to control or altogether quit the addiction. Nicotine, heroin, and alcohol have shown similar relapse rates over one year, ranging from 80% to 95% [8].

Various mechanisms can trigger relapse in drug and alcohol use, including stress, high-risk situations, failure to cope with temptation, and craving [9]. 

Several methods exist to prevent relapse from addiction to high-risk substances such as drugs, alcohol, tobacco, or gambling. These methods can be categorized into pharmacological and non-pharmacological approaches.

Pharmacological treatments work by targeting specific neurotransmitters in the brain to reduce cravings, withdrawal symptoms, and the reinforcing effects of addictive substances or behaviors. Naltrexone or acamprosate are prescribed for alcohol addiction; bupropion or varenicline for smoking cessation; and methadone or buprenorphine for opioid addiction [10,11,12,13].

Non-pharmacological approaches to relapse prevention include cognitive behavioral therapy (CBT), motivational interviewing, peer support groups, mindfulness-based relapse prevention (MBRP), psychoeducation, and holistic therapies such as yoga, acupuncture, and sound therapy. An innovative and thoroughly researched strategy involves using cutting-edge virtual reality technology to reduce the risk of relapse, revolutionizing the field of addiction intervention and prevention.

The purpose of this meta-analysis is to highlight significant new developments in the field of high-risk alcohol and drug addiction relapse, focusing on various study populations worldwide, across different age groups, and including individuals who have received pharmacological and non-pharmacological interventions during detoxification for relapse prevention.

The objectives of this paper are to explore and evaluate recent advancements in relapse prevention strategies for individuals recovering from high-risk addictions to substances such as alcohol, opioids, and illicit drugs. The research aims to identify and synthesize key findings across diverse populations and age groups, focusing on the effectiveness of pharmacological and non-pharmacological interventions in reducing relapse rates during and after detoxification.

## 2. Materials and Methods

All methodologies adhered to the guidelines outlined in the Preferred Reporting Items for Systematic Reviews and Meta-analysis (PRISMA) [14] to execute this study.

### 2.1. Data Collection

A comprehensive literature search was conducted across medical, psychiatric, and psychological databases for studies published between January 2013 and December 2023. Multiple electronic databases were systematically explored, including PubMed, Cochrane Library, Google Scholar, Semantic Scholar, and Consensus. The search strategy utilized the following key terms: ‘Addiction relapse prevention’, ’Drug relapse prevention’, and ‘Alcohol relapse prevention’, combined with the Boolean operator ’OR’ to ensure a broad retrieval of relevant studies.

The studies incorporated in the meta-analysis fulfilled the inclusion criteria:

1. Participants: studies that include patients diagnosed with alcohol use disorder (AUD) and high-risk drug addiction who were enrolled in relapse prevention programs. Participants were selected based on predefined eligibility criteria, including the severity of addiction, willingness to participate, and engagement in structured relapse prevention interventions.
2. Study Design: Studies were selected based on specific inclusion criteria, such as publication date (e.g., studies published within the last 10 years), peer-reviewed status, and language (English only). These criteria were established to ensure the inclusion of high-quality, recent, and accessible evidence. Randomized trials were prioritized to minimize bias and establish causal relationships, while the cross-sectional study provided additional insights into population characteristics and trends.
3. Intervention: Participants received various interventions, including pharmacological (e.g., medications like naltrexone or acamprosate) and non-pharmacological approaches (e.g., cognitive-behavioral therapy, motivational interviewing, and contingency management). The selection of interventions was based on their evidence-based efficacy in relapse prevention and their applicability to the target population.
4. Outcomes: The studies reported key outcomes such as gender distribution, type of addiction (alcohol vs. drug), and the effectiveness of interventions in reducing relapse rates. The primary outcome measure was the average relapse period, reported in months. Secondary outcomes included adherence to treatment, quality of life, and adverse effects of interventions.

The inclusion criteria ensured methodological rigor and relevance to the research question. Randomized clinical trials were prioritized to reduce selection bias and confounding factors. However, potential sources of bias, such as publication bias (the tendency to publish only positive results) and heterogeneity in intervention protocols across studies, were acknowledged. A comprehensive search strategy was employed to address these, including gray literature and unpublished studies where possible. Additionally, while limited in establishing causality, the cross-sectional research provided valuable descriptive data on patient demographics and addiction profiles.

### 2.2. Study Selection

**Figure 1:**
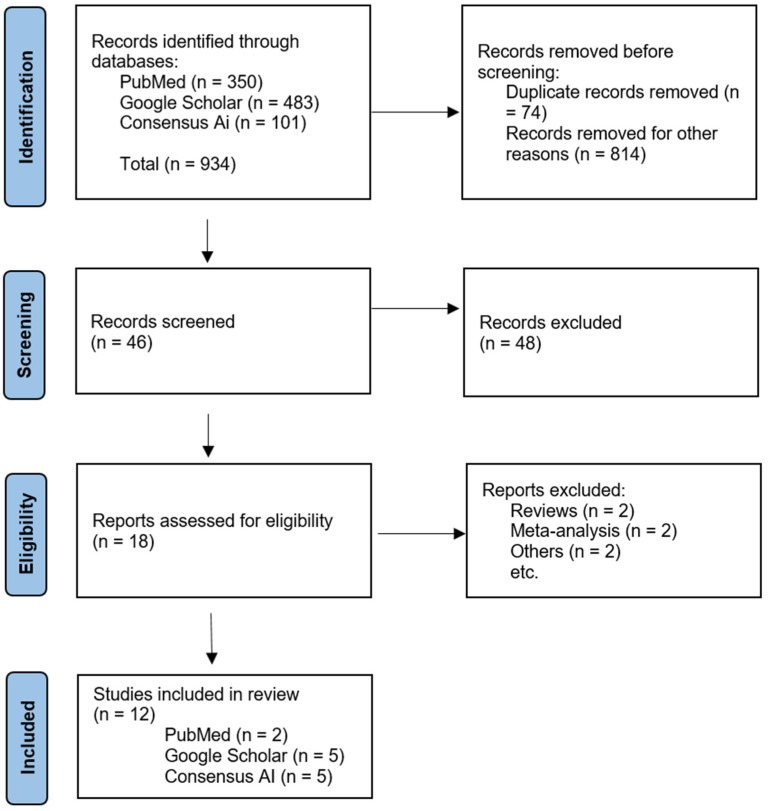
Flow diagram of preferred reporting items and the exclusion criteria.

Studies were independently assessed for inclusion based on titles, keywords, and abstracts. A workflow diagram was created to illustrate the research process for literature screening and study selection (Figure 1).

### 2.3. Data Extraction

The data were extracted as follows: country of research and year of publication, type of study, number of participants, mean age of participants, gender distribution (percentage of females and males), type of substance use issue, average relapse period of patients in each study, and the specific relapse prevention intervention used (Figure 1).

### 2.4. Data Synthesis and Analysis

The extracted data were analyzed using Python 3 in Google Colaboratory, employing libraries such as pandas, statsmodels, matplotlib, seaborn, and scipy.stats. The analysis included descriptive statistics and regression models examining relationships between the mean relapse period, average age, and gender distribution (percentage of males and females). Additionally, the study presents the results of hypothesis testing, linear regression trends over the years, and the distribution of patients based on the type of substance use. A significance level of *p* < 0.05 was considered the threshold for statistical significance in all analyses, indicating that the probability of the observed results occurring by chance is less than 5%.

## 3. Results

A workflow chart for study selection was prepared following the Preferred Reporting Items for Systematic Review and meta-analysis guidelines [14]. The titles and abstracts of 934 articles were screened; 12 studies [10,11,13,15,16,17,18,19,20,21,22,23] fulfilled all inclusion criteria and included 2162 patients. Table 1 summarizes the studies’ characteristics.

All selected studies addressed issues related to substance or alcohol abuse. The most frequently reported substances included combinations of opioids, heroin, cocaine, methamphetamine, and marijuana. The studies encompassed a wide range of pharmacological and non-pharmacological interventions, such as mindfulness-based relapse prevention (MBRP), psychoeducation, and holistic therapies.

**Figure 2:**
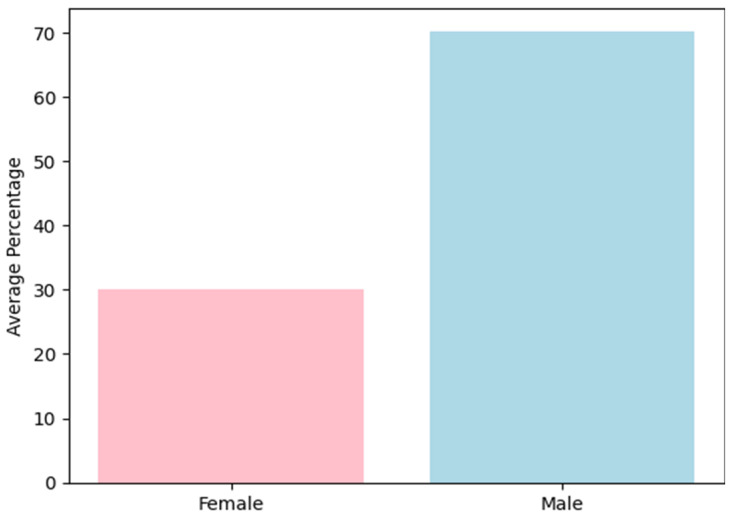
Gender distribution.

An analysis of the demographic data across the studies showed that participants ranged in age from 18 to 70 years, with a mean age of 41.43 years in the meta-analysis. Regarding gender distribution, the data reinforce the well-documented trend that addictive behaviors are more prevalent among men. Specifically, 70% of participants were male, while 30% were female (Figure 2).

### 3.1. Correlation of the Mean Period of Relapse in Studies over Other Characteristics

**Figure 3:**
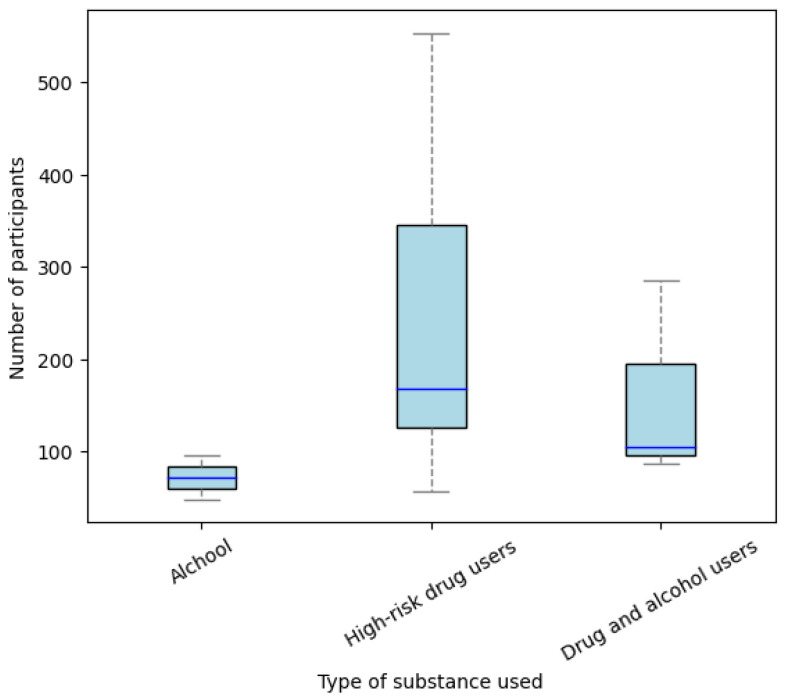
Distribution of the number of participants according to the type of substance use.

The initial line of analysis focused on determining the distribution of participants across the studies based on the type of substance use. The findings revealed that the majority of patients were high-risk drug users (Figure 3).

To analyze how different participant characteristics influence the mean relapse period, the best-fitting model, the Ordinary Least Squares (OSL) regression model, was selected based on the dataset.

The model evaluating the relationship between mean age and relapse period demonstrated an R-squared value of 0.442, indicating that age accounts for 44.2% of the variance in relapse duration—a moderate explanatory power. The associated F-statistic (8.724) and *p*-value (0.0131) confirm the statistical significance of this model at the 5% level, suggesting that age is a meaningful predictor. In contrast, gender-related models (both male and female percentages) yielded lower R-squared values (0.171 and 0.147, respectively) and non-significant *p*-values (>0.05), indicating a weaker and statistically inconclusive relationship with relapse duration. Additionally, an ANOVA test evaluating intervention type revealed a highly significant F-statistic (2.195 × 10^28^) with a *p*-value < 0.0001, emphasizing the strong impact of intervention strategies on relapse outcomes. These analyses support the conclusion that age and intervention type are the most statistically relevant predictors of relapse duration in the examined population.

In analyzing the influence of gender, an R-squared value of 0.171 was observed, indicating that the percentage of male participants explains approximately 17.1% of the variability in the mean relapse period. The F-statistic for the relationship between the male group and the relapse period was 2.264, with a corresponding *p*-value of 0.161. This suggests the model is not statistically significant at the conventional 0.05 significance level. A 95% confidence interval ([0.025, 0.975]) provides a range of plausible values for the actual population coefficient.

In the model examining the relationship between the female gender and the mean relapse period, the coefficient for the percentage of female participants was −0.0338. This indicates that the expected mean relapse period decreases by approximately 0.0338 months for each one-unit increase in the percentage of females. The confidence interval reflects the standard error [0.926, −0.025]. An R-squared value of 0.147 suggests that the proportion of female participants can explain about 14.7% of the variability in the mean relapse period. The F-statistic for this model was 1.901, with a corresponding *p*-value of 0.195, indicating that the model does not reach statistical significance at the conventional 0.05 level.

### 3.2. Effect of Interventions in Different Types of Addiction

The ANOVA test was chosen as the statistical test to evaluate the effect of different interventions on each study’s mean relapse period registration.

The sum of squares for the factor intervention type was 52.77, representing the portion of the variability in the mean relapse period explained by the different intervention categories. The F-statistic for the intervention type was approximately 2.20 × 10^28^, indicating an extremely high test statistic value used to assess the overall significance of the intervention type on the mean relapse period.

The probability associated with the F-statistic (PR(>F)) for the intervention type factor was approximately 5.26 × 10⁻^15^, indicating a highly significant result. This suggests that the likelihood of obtaining the observed F-statistic under the null hypothesis—assuming no effect of intervention type on the mean relapse period—is extremely low. The absence of an F-statistic and associated *p*-value for the residuals indicates insufficient information to assess the significance of the residual variability.

**Figure 4:**
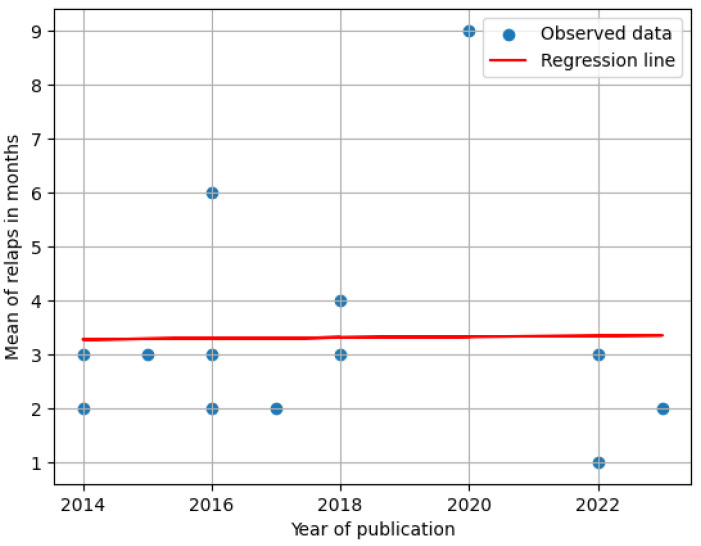
Regression of mean relapse period over the years.

Linear regression analysis of the mean relapse period across publication years did not reveal an increasing trend, suggesting that the evolution of therapeutic approaches has not significantly extended the average relapse period (Figure 4). Furthermore, a decline in research interest on relapse prevention methods was noted over the past two decades, with the majority of studies published between 2014 and 2018.

**Figure 5:**
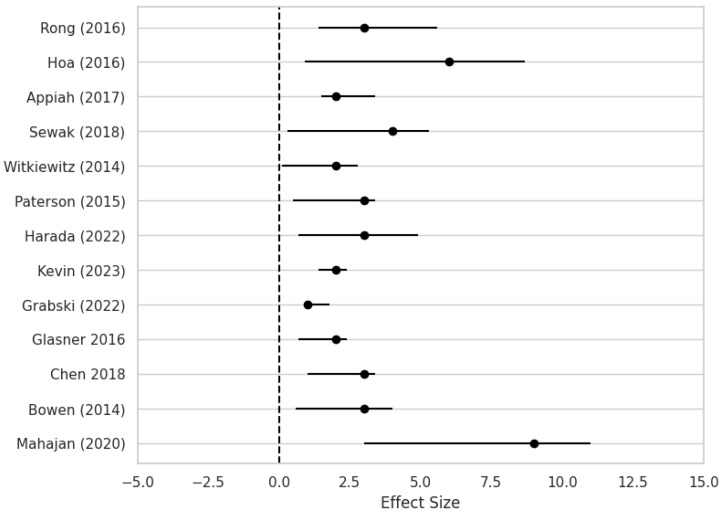
Forest plot of the mean time of relapse (months) across multiple studies [10,11,12,13,15,16,17,18,19,20,21,22,23].

The forest plot illustrates relapse outcomes across multiple studies (Figure 5). Effect sizes represent the difference in relapse duration between treatment groups, with error bars indicating the confidence intervals. Studies such as Mahajan (2020) [15] and Rong (2016) [22] reported longer relapse periods, whereas others like Glasner (2016) [18] demonstrate shorter durations. The plot highlights substantial variability in relapse outcomes across studies, suggesting possible differences in treatment efficacy or methodological approaches.

## 4. Discussion

This study conducted a comprehensive meta-analysis of 12 studies to examine key aspects of high-risk alcohol and drug addiction relapse across diverse populations worldwide, spanning various age groups and including individuals who received pharmacological and non-pharmacological interventions for relapse prevention during the detoxification phase.

The primary finding regarding the effect of mean age on relapse prevention is statistically significant, with a *p*-value of 0.0131 in the regression model. An R-squared value of 0.442 indicates that approximately 44.2% of the variance in the mean relapse period is explained by age. Notably, the studies by Gonzales (2012) [24] and Satre (2011) [25] offer valuable insights into relapse dynamics. The results suggest that younger individuals are more responsive to relapse prevention interventions for alcohol and drug addiction, highlighting the nuanced and complex nature of relapse within this demographic [24,25,26,27].

Age can influence various factors associated with relapse, including psychological resilience, comorbidities, social dynamics, and treatment responses. Understanding these can inform strategies that optimize recovery outcomes. Research shows that older adults often experience complex health profiles, frequently with higher rates of comorbidity, which can amplify the risk of relapse [28]. Young adults may respond well to technology-based solutions, such as smartphone apps that help monitor mood and provide just-in-time adaptive interventions based on behavioral triggers [29]. These technologies can effectively engage younger populations in their recovery and prevent relapses by offering real-time support and resources tailored to their needs [30].

The findings highlight that no single factor can independently predict relapse among youth [25]. While individual-level factors significantly influence the initiation and maintenance of substance use, a wide range of social and environmental influences also play a critical role in this process [31,32]. Therefore, understanding the complex interplay between personal characteristics, social dynamics, and broader environmental factors is essential for comprehending the developmental trajectories of relapse among youth undergoing treatment [24,33,34,35]. Rehabilitation has been linked to poorer outcomes over 5–9 years of consumption, particularly among individuals aged 40 and above at the study’s outset. In such cases, rehabilitation may indicate a higher risk of relapse or more severe substance-related issues within this population [25,36,37].

Emerging treatment approaches—such as virtual reality (VR) and digital medicine—offer new perspectives in relapse prevention [38,39,40]. Huang (2021) observed that VR therapy was more effective in preventing relapse among younger individuals compared to adults [41]. VR therapy enhances the sense of presence, allowing individuals to engage with simulated environments actively [41]. Digital interventions encompass a variety of strategies, including psychological therapies, cognitive function enhancement programs, and innovative technologies such as VR and biofeedback/neurofeedback. The primary appeal of digital medicine lies in its accessibility and convenience. As these technologies advance and become more widely adopted, digital medicine is expected to provide cost-effective alternatives to traditional medical services [41,42,43].

Regarding the impact of gender, the regression model suggests that a higher percentage of male participants may be associated with a longer mean relapse period; however, this effect is not statistically significant at the conventional 0.05 significance level. The model accounts for approximately 17.1% of the variability in the mean relapse period, but the overall significance remains questionable. Similarly, the model analyzing the percentage of female participants explains about 14.7% of the variance. The constant term has a coefficient of 4.324 with a standard error of 0.926. The coefficient for the percentage of females is −0.034, with a standard error of 0.025, but this result is not statistically significant (*p* = 0.195), and the overall model significance remains uncertain (*p* = 0.195).

Becker (2016) suggests that women may be more vulnerable to addiction, with a faster progression from initial use to dependence on both drugs and alcohol compared to men [44]. Additionally, women are reported to be more sensitive to the effects of stress and interpersonal difficulties in the context of alcohol addiction and relapse [44,45]. However, a 2021 review of clinical studies challenges this view, finding no consistent evidence that women are more vulnerable than men to psychostimulants, opioids, or related relapse. The available data do not support significant gender differences in craving or relapse rates [46]. On the other hand, research shows that women experience different antecedents and risks associated with substance abuse compared to men. For instance, women are more often influenced by personal relationships and social dynamics, such as stress from marriage, feelings of depression, and relationship-based substance use, which can markedly elevate their relapse potential [47,48]. Greenfield et al. emphasize that the reasons for female relapse are frequently tied to their psychosocial contexts, fundamentally differing from the external situational factors more often cited by male substance users [49]. This illustrates a need for gender-sensitive treatment approaches that consider the relational and emotional factors impacting women specifically. Moreover, studies indicate that, while women may initially engage in substance use for reasons like mood regulation and emotional coping, men are more likely to use substances for experimentation and social acceptance [50]. This fundamental difference carries through to treatment and relapse scenarios. It has been found that women are less likely to relapse after treatment compared to men, mainly when they obtain sufficient social and familial support. Yet, when they do relapse, it tends to occur in connection with intimate partner dynamics or familial stress, highlighting the intersectionality of gender and social situations in SUDs [48,51]. For instance, women often report higher levels of distress associated with family conflicts compared to men, amplifying the risk of SUD relapse. This contrasts with men’s relapse triggers, which are often tied to social factors such as living alone or peer pressure [52].

The findings highlight a clear emphasis on analyzing the distribution of participants based on the type of substance used. Notably, the results indicate a predominance of high-risk drug users within the study population. This observation calls for further exploration of how substance type may influence treatment outcomes and emphasizes the need for tailored interventions targeting this high-risk subgroup. According to the European Drug Report 2023, the most commonly consumed drug was cannabis, followed by cocaine and crack, amphetamines, heroin, and other substances [53]. Additionally, a study from the United States reported that the prevalence of individuals engaging in both alcohol and drug co-use was 5.6% [54].

Our study underscores the multifaceted nature of the factors influencing relapse periods, highlighting the need for further research into additional variables that may contribute to the observed outcome variability.

The forest plot of this meta-analysis visually summarizes individual studies’ effect sizes and confidence intervals, offering insights into the comparative effectiveness of various interventions in prolonging time to relapse. Each effect size reflects the magnitude of the difference in relapse duration between treatment groups, while the confidence intervals indicate the precision of these estimates. Notably, studies such as Mahajan (2020) and Rong (2016) exhibit larger effect sizes, suggesting substantial differences in relapse times favoring the treatment groups [15,22]. In contrast, studies like Glasner (2016) demonstrate smaller effect sizes, indicating less pronounced differences or potentially non-significant effects [18].

The variability in relapse times observed across studies may be attributed to multiple factors, including differences in study populations, intervention protocols, follow-up durations, and methodological designs [10]. Heterogeneity in patient demographics, severity of addiction, comorbid conditions, and treatment adherence can all influence relapse outcomes, contributing to the dispersion of effect sizes. Furthermore, variations in the type and intensity of interventions—from pharmacotherapy and psychotherapy to holistic or lifestyle-based approaches—may impact relapse rates and further underscore the diversity of findings across studies.

Understanding the diversity of relapse outcomes illustrated in the forest plot carries significant implications for clinical practice. Clinicians must account for the heterogeneous nature of patient populations and their varied responses to treatment when designing and implementing personalized intervention strategies [55,56,57]. Identifying interventions associated with larger effect sizes—as demonstrated in studies such as Mahajan (2020) and Rong (2016)—can guide treatment selection and optimization efforts [15,22]. Conversely, studies reporting minimal or null effects, such as Glasner (2016), highlight the need to critically assess the efficacy of existing interventions and explore alternative therapeutic approaches [18].

In addition to established pharmacological and non-pharmacological methods, increasing attention is being directed toward digital relapse prevention strategies [58,59,60]. Emerging research explores the use of virtual reality (VR) as a tool to support relapse prevention, offering unique benefits such as enhanced self-awareness, behavioral monitoring within simulated environments, and the opportunity for individuals to adopt new perspectives through avatar-based experiences [61,62,63,64,65,66]. These innovations may provide practitioners with deeper insights into the recovery process while offering patients immersive, personalized support during critical stages of relapse prevention.

One of the primary challenges associated with implementing VR interventions in mental health and rehabilitation is the requirement for significant resources, including financial investment, infrastructure, and trained personnel [67,68]. Despite its promise, developing high-quality VR applications necessitates substantial time and expertise, which can delay deployment within clinical settings. Furthermore, practitioners often must navigate the complexities of patient training and familiarization with VR tools, which can hinder immediate effectiveness. These challenges are compounded by the evolving nature of VR technology, which may lead to frequent updates and modifications, creating an additional burden for healthcare providers who wish to effectively incorporate these innovations into their practices. Another critical challenge is the ethical and clinical validation of VR applications. As VR technologies advance, questions regarding informed consent, data privacy, and the potential for unintended psychological effects during exposure to virtual environments become essential. For VR therapies targeted at treating conditions like PTSD or anxiety disorders, clinicians must ensure that exposure techniques do not retraumatize patients, particularly in vulnerable populations [69,70]. Additionally, ensuring robust safety protocols for monitoring patient reactions in a VR setting is imperative, though the immersive nature of the technology may inadvertently detract from direct human interaction.

Additional studies on alcohol relapse prevention and craving have provided valuable insights into the effectiveness of combining VR interventions with CBT [71,72,73,74,75]. VR represents a novel technique that complements traditional treatment approaches and has shown the potential to elicit cravings through controlled exposure to alcohol-related environments. However, while promising, the superiority of VR in assessment and relapse management still requires further empirical validation [75]. High-fidelity simulations offer potential therapeutic benefits but also pose challenges, including the risk of overstimulation or triggering. Nevertheless, the VR approach is a powerful tool for developing personalized interventions, marking a promising frontier in psychiatry and psychology [76,77].

The limitations of this meta-analysis include the relatively small number of studies available in this field, the inherent challenges of enrolling individuals with addiction into clinical trials, and the limited quality and consistency of data reported in the included studies.

This meta-analysis is subject to limitations, including potential publication bias and methodological heterogeneity across the included studies, which may affect the generalizability and consistency of the findings.

We have noted a reduction in relapse prevention research output since 2018. This downturn may stem from various overlapping causes, such as evolving focus areas within addiction science, financial constraints limiting support for long-term studies, and increasing ethical or regulatory hurdles—especially when working with high-risk populations. Furthermore, challenges in maintaining participant engagement and continuity throughout studies can impede reliable data gathering. These issues point to an underexplored field that merits deeper examination to better understand its consequences for developing effective strategies to prevent relapse. Future research should move beyond basic demographic profiling to explore the complex interplay between intervention type, social determinants, and individualized treatment needs. Integrating these multidimensional factors into large-scale randomized controlled trials could yield more nuanced insights into relapse prevention and contribute to improved outcomes for diverse populations affected by substance use disorders.

## 5. Conclusions

This meta-analysis highlights that, while age emerged as a statistically significant predictor of relapse duration, it should not be viewed in isolation. Our findings indicate that intervention type—mainly the distinction between pharmacological and non-pharmacological methods—is essential in influencing relapse outcomes, as demonstrated by highly significant ANOVA results. Interventions such as mindfulness-based relapse prevention (MBRP), cognitive behavioral therapy, and emerging digital tools like virtual reality have shown promising variability in effectiveness, suggesting that tailored treatment approaches may enhance long-term recovery. The influence of gender in relapse prevention appears to be multifaceted, with current evidence suggesting that, while statistical significance remains limited, gender-specific psychosocial factors may influence shaping relapse risk and treatment responsiveness. Additionally, although not directly measured in all studies, the impact of social and environmental factors—such as family support, peer influence, and gender-specific psychosocial dynamics—warrants more profound attention. These contextual variables, often underrepresented in statistical models, may mediate or moderate the effectiveness of clinical interventions and should be considered essential elements in designing relapse prevention strategies.

## Figures and Tables

**Table 1:** Characteristics of studies.

Study	Country	Year	Study Type	No. Participants	Mean Age	Percent Male	Percent Female	Substance Use Issue	Mean Relapse Period (Months)	Intervention Type	Effect Size/Key Findings	Follow-Up Duration
Bowen S. [16]	USA	2014	RCT	286	54	71.50%	42.10%	drug use, heavy drinking	3	Mindfulness-Based Relapse Prevention (MBRP), Relapse Prevention (RP), Treatment As Usual (TAU)	MBRP led to significantly fewer days of substance use and heavy drinking at 12-month follow-up vs RP and TAU; effect sizes not explicitly provided	12 months
Chen X. [17]	China	2018	RCT	180	36.5	83%	17%	methamphetamine	3	MBRP + Virtual Reality Cue Exposure (VRCE), MBRP alone, Treatment As Usual (TAU)	Study protocol only; no outcome data or effect sizes available yet	3 and 6 months planned
Glasner S. [18]	USA	2016	RCT	63	45.3	71.40%	28.60%	stimulants	2	MBRP + Contingency Management (CM) vs Health Education + CM	Medium effect sizes for reduced depression (d=0.58) and psychiatric severity (d=0.61); lower odds of stimulant use in MBRP group (OR=0.78 for depression, OR=0.68 for anxiety)	1 month post-treatment
Grabski M. [12]	UK	2022	double blind clinical trial	96	44.07	53.54%	36.46%	alcohol use	1	Ketamine infusions (with or without MBRP) vs placebo infusions (with or without alcohol education)	Ketamine + therapy group had 15.9% more abstinent days vs control (95% CI: 3.8%, 28.1%) at 6 months; well tolerated	6 months
Lynch K.G. [13]	USA	2023	double blind clinical trial	156	51	78%	22%	cocaine use	2	Varenicline + Cognitive Behavioral Therapy (CBT) vs Placebo + CBT	No significant differences in cocaine abstinence, craving, or withdrawal symptoms between groups	12 weeks
Harada T. [19]	Japan	2022	RCT	48	53.3	75%	25%	alcohol use	3	CBT-based Relapse Prevention (RP) vs Psychoeducation (PE)	No significant differences between RP and PE groups in relapse rate or psychological measures	3 and 6 months
Paterson L. [20]	UK	2015	RCT	87	42.5	81%	19%	alcohol, opiate, cocaine	3	Pharmacological (naltrexone, GSK598809, aprepitant) in experimental medicine study with fMRI	Study focused on feasibility and brain response; no clinical relapse outcome or effect size reported	Not applicable
Witkiewitz K. [21]	USA	2014	RCT	105	35.8	0%	100%	methamphetamine, heroin, cocaine, alcohol, marijuana, nicotine	2	Mindfulness-Based Relapse Prevention (MBRP) vs Relapse Prevention (RP)	MBRP group had fewer drug use days and fewer legal/medical issues at 15-week follow-up	15 weeks
Sewak R. [11]	USA	2018	RCT	116	40	62.93%	37.06%	drugs use	4	Sound-based auditory stimulation (binaural beats, music, subliminal messages)	Preliminary hypothesis and early RCT suggest sound may reduce relapse risk; no standardized effect size provided	Not specified
Appiah R. [23]	Ghana	2017	clinical trial	15	43.5	86.60%	13.30%	drugs use	2	Multilevel relapse prevention strategies: clinical, spiritual, social, individual	Qualitative findings suggest contextual and spiritual strategies enhance recovery in Ghana	1 year (post-treatment interviews)
Vo H.T. [10]	USA	2016	clinical trial	56	23.1	70%	30%	opioid use	6	Buprenorphine or Extended-Release Naltrexone (XR-NTX)	Retention ~65% at 12 weeks, 40% at 24 weeks; no significant differences between medications in opioid abstinence	24 weeks
Rong C. [22]	China	2016	RCT	554	41.6	80%	20%	heroin use	3	Methadone or Jitai tablets with psychological counseling and social support	Psychological counseling significantly reduced relapse (OR = 3.56); longer drug history increased relapse risk	2 years

## Data Availability

No new data were created or analyzed in this study.
